# *Lactiplantibacillus plantarum* Z45 from Sour Soup Improves Flavor and Safety of Fermented Corn: Insights from Genomic and Metabolomic Approaches

**DOI:** 10.3390/foods14213803

**Published:** 2025-11-06

**Authors:** Mengdi Zhao, Yuanyuan Zhang, Yi Wu, Shuang Liang, Guangyu Li

**Affiliations:** 1 College of Animal Science and Technology, Qingdao Agricultural University, Qingdao 266109, China; 19950807@mails.jlau.edu.cn (M.Z.); 20222103021@stu.qau.edu.cn (Y.Z.); 20232109046@stu.qau.edu.cn (Y.W.); 20232209008@stu.qau.edu.cn (S.L.); 2 College of Animal Science and Technology, Shandong Agricultural University, Taian 271018, China

**Keywords:** fermented corn, *Lactiplantibacillus plantarum*, whole-genome sequencing, non-targeted metabolome, fermentation properties, food safety

## Abstract

Sour soup, a traditional fermented specialty from Northeast China, is renowned for its distinctive aroma and various health benefits. Here, we report the probiotic properties of *Lactiplantibacillus plantarum* Z45—a strain isolated from sour soup broth—along with its fermentative potential in sour soup production. This strain is suitable for food fermentation due to its absence of biogenic amine production and non-hemolytic activity. It exhibited strong tolerance to simulated gastrointestinal conditions and demonstrated high adherence capability to Caco-2 cells. Additionally, the strain displayed antioxidant and antimicrobial activities. Whole-genome sequencing revealed that Z45 carries no virulence or antibiotic resistance genes. It also harbors multiple carbohydrate-active enzymes and a complete folate biosynthesis pathway, alongside genes associated with stress response, antioxidant activity, and adhesion. Subsequently, Z45 was employed as a starter culture for sour soup fermentation, and its effects on the physicochemical and sensory properties of the product were evaluated. The results indicated that fermentation with Z45 did not alter the physicochemical properties of sour soup but significantly enhanced its sensory attributes. Compared to spontaneous fermentation, Z45-fermented sour soup showed reduced levels of harmful biogenic amines, improved flavor and overall sensory quality, notable enrichment of *Lactobacillus* and *Oscillospirales* in the microbial community, and upregulation of beneficial metabolites such as flavonoids and glycerophosphocholine. In summary, *Lactiplantibacillus plantarum* Z45 is safe, demonstrates probiotic potential, and holds promise for improving the quality and functional properties of fermented foods.

## 1. Introduction

Research on probiotics has gained momentum due to increasing health awareness and concerns. The development of next-generation probiotics is considered an important strategy for regulating gut flora and improving human health [1]. Probiotics are defined as live microorganisms that upon ingestion in sufficient quantities could confer health benefits [2]. Recent studies highlighted the potential health benefits of fermented properties as a source of probiotics in the human diet [3], where they are involved in the modulation of the immune system, interactions with the host and its gut flora, improvement of the intestinal barrier, and production of enzymes and small molecules with systemic positive effects [4]. Previous investigations on probiotics were primarily focused on strains of human origin [5,6]. However, there is growing interest in identifying new strains from local fermented foods, especially those that remain unexplored, to uncover novel resources that promote improved host health and address gut dysbiosis [7].

Sour soup is a traditional fermented corn-based food with distinctive Manchu ethnic characteristics. Recognized for its tangy sourness and smooth texture, it remains a staple food staple in Northeast China [8]. Previous studies have investigated the cultivability of beneficial microorganisms involved in the fermentation process of sour soup, providing a foundation for developing defined starter cultures to facilitate its industrial-scale production. Furthermore, lactic acid bacteria derived from traditional Chinese fermented foods like sour soup represent a significant source of functional microbes [9]. The naturally fermented corn product “sour soup” may have some safety concerns, such as the presence of harmful microorganisms that produce harmful metabolites, including biogenic amines (BAs), with *Burkholderia gladioli pathovar cocovenenans* being a notable example of a serious threat to human health [10]. However, as an acidic fermented product, it is reasonable to expect the product to have lactic acid bacteria (LAB) that potentially act as probiotics. Traditionally, the safety and probiotic potential of strains were assessed through their physiological and biochemical characteristics. However, with the increasing prevalence of high-throughput sequencing technology in recent years, a shift towards studying strains by focusing on molecular mechanisms and genetic characterisation has been evolving [11]. Whole-Genome Sequencing (WGS) has the advantage of being rapid and low-cost to annotate gene function in microorganisms [12]. Detailed information about metabolites could be provided by metabolomics, which allowed for a more thorough molecular picture of food components, and thus facilitated an effective assessment of food properties [13]. Therefore, it is crucial to employ sequencing methods to analyse genes associated with metabolites and probiotic activity [14].

This study systematically investigated the effects of *L. plantarum* Z45 as a starter culture on the quality attributes of sour soup. Furthermore, high-throughput sequencing and non-targeted metabolomics were employed to characterize the bacterial community structure and metabolic profiles. The findings offer valuable insights for enhancing the quality and flavor of this traditional snack.

## 2. Materials and Methods

### 2.1. Isolation of Strains

The samples were primarily collected from Baicheng City and Liaoyuan City in Jilin Province. Traditional Chinese sour soup is a fermented food produced through natural fermentation. The conventional preparation process is as follows: After 500 g of corn is thoroughly washed, it is placed into a 5 L sealed culture container. Distilled water is added at a ratio of 1:4 (*V*:*V*), and the mixture is stored in a cultivation chamber. Following a 21-day fermentation period, the corn is removed and, together with a portion of the fermentation broth, ground into a paste (“soup base”) using a soymilk maker. It was at this stage that sampling was conducted in the present study for bacterial isolation.

Traditional sour soup (5.0 g) was diluted with sterile saline in an enzyme-free and sterile 50-mL centrifuge tube and mixed using a vortex mixer (Vortex-5S, Huxi, China) for 5 min. Subsequently, a series dilution was performed with the sterile saline, and 100 μL of the diluted solution was applied to Man, Rogosa, and Sharpe (MRS) plates (Haibo Co., Qingdao, China). Invert the plate coated with diluted bacterial solution into the incubator and incubate at 37 °C for 48–72 h. Individual colonies were obtained for four rounds of purification.

### 2.2. Antibacterial Activity

The isolates were added to each well of tryptone soy agar (TSA) using the Oxford Cup bilayer method, with uninoculated MRS culture serving as a blank control. The isolates were harvested after 24 h of incubation in MRS liquid medium. The concentration of the bacterial solution was adjusted to approximately 1 × 10^7^ CFU/mL. The antibacterial activity was tested against pathogenic bacteria (*Escherichia coli* ATCC 25922, *Staphylococcus aureus* ATCC 25923, *Salmonella* ATCC 14028, *Pseudomonas aeruginosa* ATCC 27853, *Listeria monocytogenes* ATCC 19115, and *Streptococcus pneumoniae* ATCC 49619) that were cultured in Luria–Bertani (LB) liquid medium to stabilise growth. All plates were subsequently incubated at 37 °C for 24 h. The concentration of pathogenic bacteria was adjusted to the same concentration as that of the isolated strain. Finally, the inhibition zone diameter (IZD) was measured. Each experimental group was performed in triplicate.

### 2.3. Identification of Strains

Detection of isolated strains by Gram staining. Gram-positive strains were then tested for catalase activity using the hydrogen peroxide test [15]. The presence of bubbles within a 30-s timeframe indicated a positive result for catalase activity.

### 2.4. Growth Curves of Isolated Strains

Cultures grown for 24 h were inoculated into MRS liquid medium at 37 °C at an inoculum rate of 3.0% (*v*/*v*). The absorbance at OD_600nm_ was measured using 200 µL of the bacterial solution.

### 2.5. Evaluation of Probiotic Potential

The probiotic effects of the isolated microorganisms were evaluated using the methodology described by Zhao et al. [16].

#### 2.5.1. Antioxidant Activity

The determination of 2,2-Diphenyl-1-picrylhydrazyl (DPPH), 2,2′-Azinobis-(3-ethylbenzthiazoline-6-sulphonate) (ABTS) free radical scavenging, and superoxide anion scavenging activities was conducted using previously reported methods [16].

#### 2.5.2. Bile Salt Resistance and Acid Resistance

Bacterial cultures were grown for 24 h, centrifuged at 4 °C at 10,621× *g* for 10 min and washed twice with phosphate-buffered saline (pH 7.0). The bacterial pellets were resuspended in equal volumes of 0.1~0.5% (*w*/*v*) bile salt solutions with a variation gradient of 0.1%. The acid resistance test is similar to the treatment above. The bacterial pellets were resuspended in an equal volume of solutions with pH values of 1.5, 2.0, 2.5, 3.0, 3.5, and 4.0. Samples were collected at 0, 2, 4, 6, and 8 h of mixing to measure absorbance at OD_600_ nm.

#### 2.5.3. Gastrointestinal Fluid Tolerance, Auto-Aggregation Activity and Cell Surface Hydrophobicity

After incubation in MRS medium for 24 h, the bacterial pellets of the isolated strain were washed with PBS (Phosphate-Buffered Saline, pH 7.0) and then resuspended in simulated gastric fluid (Solarbio, Beijing, China). After a 3 h incubation period, the proteins were washed with PBS and resuspended in simulated intestinal fluid (Solarbio, Beijing, China), followed by a 4 h incubation period. The survival rates of the isolates in the simulated gastric (0 h and 3 h) and intestinal fluids (0 h and 4 h) were calculated using the dilution smear counting method [17]. Auto-aggregation activity and cell surface hydrophobicity were assayed with reference to the previous methods of the laboratory [18].

#### 2.5.4. Detection of Adhesion to Caco2 Cells and Folate Content

The Caco-2 cell line was obtained from Procell Life Science & Technology Co., Ltd. (Wuhan, China). Referring to the previously described method [19], the cells were seeded in 12-well plates at a density of 1 × 10^5^ cells per well. When the cell confluence reached approximately 90%, the Caco-2 monolayers were washed with PBS to remove antibiotics. Subsequently, 1 mL of bacterial suspension (1 × 10^8^ CFU/mL) was added to each well, followed by co-incubation for 90 min at 37 °C in a 5% CO_2_ atmosphere. Finally, Triton X-100 was added to lyse the cells and release the adherent bacteria. Each experiment was performed in triplicate and independently repeated at least three times. The adhesion rate was calculated as follows:

Adhesion rate (%) = (CFU of adherent bacteria/CFU of initially added bacteria) × 100%

The folate content in the bacterial supernatant was quantified using a folate ELISA kit (Meilian, Shanghai, China) according to the manufacturer’s instructions. All samples were measured in triplicate.

### 2.6. WGS and Annotation of Z45

After a 24 h incubation period, the bacterial pellets were collected. Bacterial DNA was extracted using a DNA kit (Qiagen, Beijing, China). Following sample detection and purification, a library was constructed using the SQK-LSK110 kit (Oxford Nanopore Technologies, Oxford, UK) according to the manufacturer’s instructions, while a short-fragment library was prepared using the VAHTS^®^ Universal Plus DNA Library Prep Kit for MGI V2/for Illumina V2 (Vazyme, Najing, China). After passing quality control, whole-genome sequencing was performed on the Illumina NovaSeq 6000 platform [18]. The final assembly resulted in a closed genome of the Z45 strain for downstream analysis. The databases and web sites used for the functional analyses are listed in Supplementary Table S1.

### 2.7. In Vitro Evaluation

#### 2.7.1. Haemolytic Activity

The activity was determined as described by Adimpong et al. [20]. In brief, the Z45 bacteria was streaked onto tryptone soy agar with sheep blood (TSA-SB, Oxoid Ltd., Wesel, Germany) and incubated at 37 °C for 24 h. Uninoculated blood agar plates were used as negative controls, while *S. aureus* (ATCC 25923) was used as a positive control.

#### 2.7.2. Antibiotic Susceptibility Assay

The antibiotic susceptibility profile was assessed using the disc diffusion method [21] with minor modifications. The isolates were inoculated in Mueller–Hinton (MH) broth (Haibo, Qingdao, China) and incubated at 37 °C until they reached a turbidity of 0.5 McFarland’s standard. The suspension was then inoculated onto MH plates using cotton swabs, and antibiotic paper discs (Penicillin, Ampicillin, Imipenem, Gentamicin, Minocycline, Doxycycline, Chloramphenicol, Erythromycin, Clindamycin and Azithromycin) were promptly applied to the agar plates. After 24–48 h of incubation at 37 °C, the IZD was measured.

### 2.8. Experimental Design for Sour Soup Fermentation with Bacterial Strains

The operational procedure for fermenting sour soup was consistent with the process described in Section 2.1. A control group (CK) group was allowed to ferment naturally. A Z45 group was inoculated with 1 × 10^8^ CFU/mL of Z45 pure culture. A Z15 group was inoculated with 1 × 10^8^ CFU/mL of Z15 pure culture. Group M was a mixed group, with the amount of each strain added according to Z15:Z45 = 1:1, while keeping the total amount of strains added unchanged. After 3 weeks, samples were taken and quickly frozen in liquid nitrogen.

### 2.9. Sensory Evaluation, Safety Assessment and Physicochemical Parameters

Prior to sensory evaluation, the ground paste from the natural fermentation group, the *L. plantarum* Z45 group, and the Z15 group was pressed into noodle-like strands using a perforated ladle and cooked directly in boiling water. The resulting edible sour soup was then used for sensory evaluation.

#### 2.9.1. Sensory Evaluation

A sensory panel consisting of twelve trained evaluators was established to assess the sour soup samples. All evaluations were conducted following the criteria detailed in Supplementary Table S2. A double-blind protocol was implemented to minimize bias: a researcher not involved in the tasting session prepared all samples and assigned them random three-digit codes generated using the Randbetween (100,999) function in Excel. This researcher maintained the exclusive code-sample identity key. Another team member, who was also unaware of the sample identities, distributed the coded samples according to a pre-randomized sequence. Panelists were kept blind to sample groupings and were prohibited from communicating with each other during sessions. They individually rated each sample based on the provided score sheet. Final scores represent the mean values derived from all panelists’ ratings across the three replicates. Data were analyzed using one-way ANOVA, with statistical significance set at *p* < 0.05. The experiment was independently repeated three times.

#### 2.9.2. Biogenic Amine Detection

A 50 μL sample aliquot was combined with 100 μL of sodium bicarbonate solution, 10 μL of sodium hydroxide, and 100 μL of dansyl chloride solution, followed by thorough vortex mixing. Derivatization was carried out in a water bath at 60 °C for 25 min. After cooling to room temperature, 10 μL of ammonia was added. The mixture was then evaporated to dryness and reconstituted in 1 mL of acetonitrile. The resulting solution was filtered through a 0.22 μm membrane, and 3 μL of the filtrate was injected into the LC-MS/MS system [22]. Separation was achieved using a HYPERSIL GOLD C18 column with a mobile phase consisting of (A) 0.1% (*v*/*v*) formic acid in water and (B) acetonitrile, at a flow rate of 0.3 mL/min and a column temperature of 35 °C. For all eight biogenic amines, the recovery rates were all above 85.0%. The limits of detection (LOD) were determined to be between 0.05 and 0.21 mg/L, while the limits of quantification (LOQ) ranged from 0.10 to 0.3 mg/L.

#### 2.9.3. Physicochemical Parameters

The pH value of the fermented sour soup was measured at room temperature using a pH meter (PHS-3E, Leici, Shanghai, China). The determination of titratable acidity (TA) was performed according to a previously described method [23].

### 2.10. 16S rRNA Sequencing

Genomic DNA was extracted using a DNA kit (Omega Bio-Tek,, Norcross, GA, USA), and libraries were sequenced on the NovaSeq 6000 platform (Illumina, San Diego, CA, USA) [18]. The V_3_-V_4_ region was amplified with the specific primers 515 F (5′-GTGYCAGCMGCCGCGGGTAA-3′) and 806 R (5′-GGACTACNVGGGTWTCTAAT-3′) containing barcodes, under the following conditions: 95 °C for 4 min, 61 °C for 1 min, 72 °C for 2 min, repeated for 30 cycles. The exact same DNA extraction procedure was performed using sterile nucleic acid water instead of samples to exclude DNA contamination. Libraries were prepared using the TruSeq Nano DNA LT Library Prep Kit (Illumina, San Diego, CA, USA). Total RNA/DNA was subjected to sequencing on the Illumina NovaSeq 6000 platform, which generated 2 × 250 bp paired-end reads. Data quality testing was performed at Q20 > 98% and Q30 > 95% and passed for downstream analysis. Raw sequencing data obtained were processed using cutadapt software to remove primer sequences from the raw data sequences, and then QIIME2 (version: 202202) was used for quality filtering, noise reduction and splicing to obtain representative sequences and ASV abundance tables. The original data presented in the study are openly available in NCBI under the accession number PRJNA1335621. Subsequent analyses were visualised using the R package (4.2.1) and chiplot (https://www.chiplot.online/; accessed on 15 April 2025).

### 2.11. Non-Targeted Metabolomics Analysis of Sour Soup

The extraction and analysis of metabolites were carried out as described by Zhao et al. [24]. Chromatographic separation was performed on an ACQUIY UPLC BEH Amide column (2.1 mm × 100 mm, 1.7 µm; Waters, Ireland, MA, USA) under HILIC mode. The mobile phase consisted of (A) 25 mM ammonium acetate in ammonia water and (B) acetonitrile. The gradient elution program was set as follows: 95% B (0–0.5 min), decreased linearly to 65% B over 6.5 min, then to 40% B over 1 min and held for 1 min, followed by a rapid increase to 95% B in 0.1 min and re-equilibration for 2.9 min. MS analysis was conducted on an Orbitrap Exploris™ 480 mass spectrometer (Thermo Scientific, San Jose, CA, USA) equipped with an ESI source. The source parameters were as follows: curtain gas (Gas1), 50 arbitrary units; nebulizing gas (Gas2), 60 arbitrary units; ion source temperature, 350 °C; and spray voltage, 3500 V in positive mode and 2800 V in negative mode. Full-scan data were collected from *m*/*z* 70 to 1200 at a resolution of 60,000, with an accumulation time of 100 ms and dynamic exclusion of 4 s. Raw data were processed using R. Univariate analysis was performed using Student’s *t*-test to evaluate intergroup differences. Significantly altered metabolites were selected based on variable importance in projection (VIP) > 1.0 and *p* < 0.05. Multivariate analyses, including PCA and OPLS-DA, were applied to these metabolites. Model robustness was assessed via 7-fold cross-validation and permutation testing. The false discovery rate (FDR) was controlled using the Benjamini–Hochberg procedure, with FDR-adjusted *p* < 0.05 considered statistically significant. Differential metabolites were further subjected to KEGG pathway enrichment analysis (http://www.kegg.jp/; accessed on 10 April 2025), with FDR-corrected *p* < 0.05 indicating significant enrichment.

### 2.12. Statistical Analysis

All experiments were repeated three times. The data are expressed as the mean ± standard deviation (SD). Statistical significance was determined using *t*-tests and one-way analysis of variance in GraphPad Prism 8.3.0. Results were significant at *p* < 0.05.

## 3. Results and Discussion

### 3.1. Antibacterial Activity of Isolated Strains

The ability to inhibit pathogens’ growth is an essential criterion for evaluating potential probiotic candidates [25]. A total of 33 strains were isolated from sour soup, including 17 Gram-positive and catalase-negative bacterial strains (Supplementary Figure S1A). Among these, the top five strains exhibiting the strongest antibacterial activity were selected for further assessment of their antioxidant properties (Supplementary Figure S1B–D). Among them, *L. plantarum* Z45 demonstrated the strongest antibacterial effect, surpassing that of several previously reported *L. plantarum* from dairy and fermented sources [26]. In addition, we conducted a preliminary verification of the effective antibacterial metabolites and found that after neutralizing organic acids, the antibacterial effect was significantly affected, whereas proteases and high temperatures had little impact. Therefore, we speculate that the main mechanism by which the strains inhibit pathogenic bacteria is through the production of organic acids. Probiotics have been reported to maintain intestinal homeostasis by adhering to and colonising the host intestine, thereby exerting antioxidant effects. In addition, probiotic metabolites can mitigate oxidative damage, thereby slowing down damage [27,28]. Overall, the results reveal strains Z15 and Z45 exhibited superior antioxidant capacity compared to other strains.

### 3.2. Probiotic Potential Testing Results of the Strain

**Figure 1:**
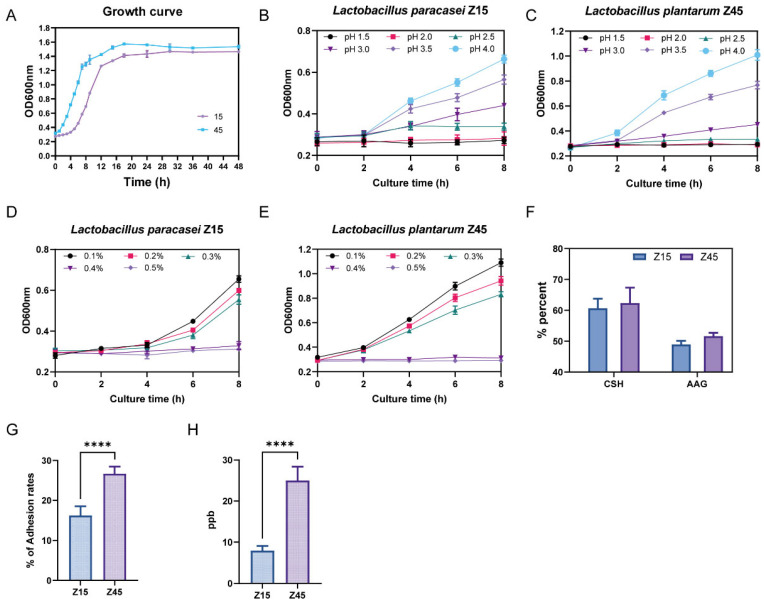
In vitro evaluation of probiotic potential. (**A**) Growth curve; (**B**) growth curves of Z15 at different pH values; (**C**) growth curves of Z45 at different pH values; (**D**) bile salt resistance of Z15; (**E**) bile salt resistance of Z45; (**F**) auto aggregation ability and cell surface hydrophobicity; (**G**) adhesion rates of Caco2 cells; and (**H**) folic acid content. **** indicate that *p* < 0.0001.

Based on the bacteriostatic and antioxidant results, strains Z15 and Z45 were selected to evaluate their in vitro probiotic potential. Tolerance to the gastrointestinal environment is essential for probiotics’ functionality. The most critical factors are their tolerance to the highly acidic environment of the stomach and the bile salt concentration in the small intestine [29]. The trends of the growth curves of the two strains were basically the same (Figure 1A). Strain Z45 demonstrated greater effectiveness in tolerating low pH and bile salts compared with strain Z15 (Figure 1B–E). Further analysis showed that strain Z45 exhibited significantly better tolerance to artificial gastrointestinal fluids than strain Z15 (*p* < 0.05, Table 1). In contrast, strain Z15 showed significantly better tolerance to intestinal fluid than strain 45 (*p* < 0.05). No significant difference was observed in tolerance between the two strains when considering the combined results of artificial gastroenteritis (*p* > 0.05). Notably, strains Z15 and Z45 were more tolerant to enteric fluids compared with the previously reported *L. plantarum* 9010 and DB9, isolated from fermented pickles, showing better potential functionality in the host GIT [30]. Another desired characteristic of probiotics is their ability to adhere to the host’s enterocytes. Adhesion is required for colonisation and for direct interactions between the probiotic and host cells, which leads to the competitive exclusion of pathogens and/or modification of host cell responses [31]. This adhesion capacity can be indirectly assessed by measuring hydrophobicity and self-aggregating activity [32]. No significant difference (*p* > 0.05) was observed in the in vitro adhesion capacity between the two strains (Figure 1F). Since probiotics are designed to colonize and adhere to the host intestinal tract, one of their most critical characteristics is the ability to attach to mucosal cells. This adhesion is essential for sustaining the viability of lactic acid bacteria (LAB) within the gastrointestinal environment [33]. The adhesion capability of strain Z45 to Caco-2 cells was significantly higher than that of strain Z15 (Figure 1G, *p* < 0.05), and was superior to the *L. plantarum* strains from fermented pickles previously reported in the literature. [34]. Furthermore, based on whole-genome prediction, we additionally performed folate content assays and found that the folate production capacity of Z45 was significantly greater than that of Z15 (Figure 1H, *p* < 0.05), which is consistent with the genomic prediction.

### 3.3. WGS and Annotation

**Figure 2:**
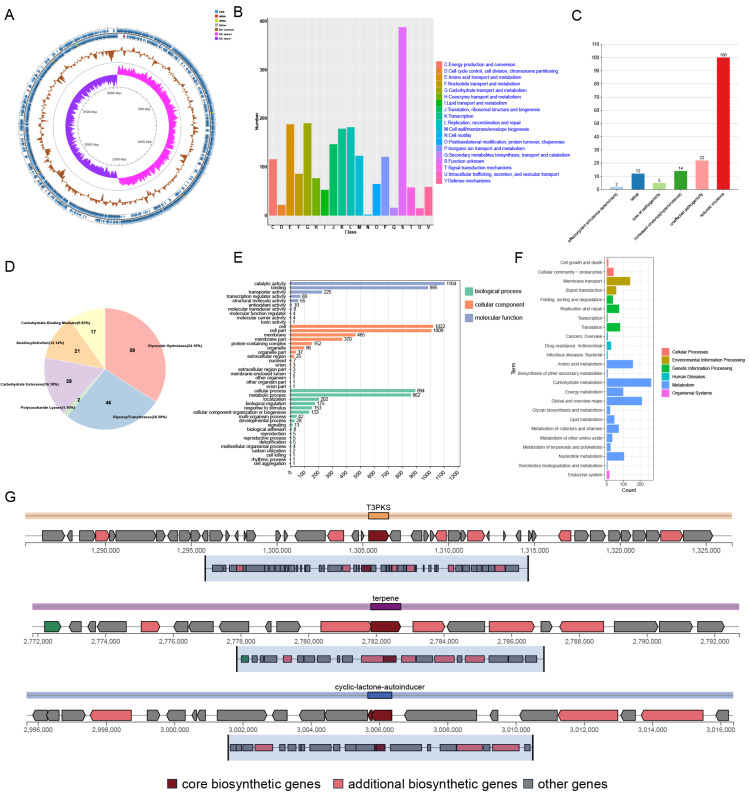
Genome-wide annotation and analysis of *L. plantarum* Z45. (**A**) Genome map; (**B**) COG functional annotation; (**C**) Pathogen–Host Interaction annotations; (**D**) Carbohydrase annotations; (**E**) GO functional annotation; (**F**) KEGG functional annotation and (**G**) AntiSMASH predicts bacteriocin of *L. plantarum* Z45.

The strain with the highest probiotic potential, Z45, was selected for further analysis. Initially, strain Z45 was streaked on MRS plates, where it appeared white, round, smooth, Gram-positive, and rod-shaped (Supplementary Figure S2A–C). A genome-wide analysis identified strain 45 as *L. plantarum* (Figure 2A, Supplementary Table S3), with a genome size of 3.05 Mb, 2923 genes, and a GC content of 44.89%. The observed genome size is consistent with existing data on *L. plantarum* strains isolated from food sources [35]. The Z45 genome was annotated using Rapid Annotation using Subsystem Technology, revealing the presence of 60 insertion sequences (Supplementary Table S4), which may contribute to genetic diversity [36]. Additionally, the *L. plantarum* Z45 genome contains a prophage (Supplementary Table S5), which could improve strain fitness and contribute to the release of extracellular membrane vesicles [37]. This implies that the prophage may confer benefits such as increased resistance to environmental stressors, enhanced survival mechanisms, or competitive advantages. Additionally, the presence of the prophage may be linked to the production of extracellular membrane vesicles, which are known to participate in various biological processes, including intercellular communication, nutrient acquisition, and stress response, potentially contributing to the strain’s overall functionality and ecological success.

To assess the safety of strain Z45 for food applications, the strain’s genome was analyzed for the presence of antimicrobial resistance genes, virulence factors, and its potential to become a human pathogen. It was determined by the CARD analysis that *L. plantarum* Z45 was free of antimicrobial resistance genes. Eight virulence factors were identified, all associated with immune response, adhesion, and stress-related genes (Table 2). The virulence genes *groL* and *tufA* were present in the *L. plantarum* Z45 genome; however, these genes are believed to be associated with the survival and colonisation of probiotics and are no longer regarded as virulence factors [38]. Thus, it was postulated that the Z45 genome did not contain any genes linked to the synthesis of harmful compounds. Notably, its low likelihood of being a human pathogen further suggests that it is safe for use in food fermentation processes with *Lactobacillus* [17].

Further analysis of the data revealed that the whole-genome COG of *L. plantarum* Z45 was most frequently annotated with unknown functions (388, Figure 2B), consistent with the findings of a previous study on *L. plantarum* DMDL 9010 [39]. This was followed by annotations associated with carbohydrate transport and metabolism (190). As shown in Figure 2C, *L. plantarum* Z45 was also annotated with reduced virulence. Notably, Z45 was observed to possess 173 carbohydrate enzymes, with glycoside hydrolases being the most abundant (Figure 2D, 59, 34.10%). *L. plantarum* typically contains between 90 and 119 CAZymes [40]. Z45 contains many CAZymes, including 46 Glycosyl Transferases (GTs) and 59 Glycoside Hydrolases (GHs). Given their capacity to hydrolyse complex carbohydrates, GHs are believed to be the primary enzymes responsible for the metabolism of carbohydrates by gut flora. Also, GTs play a crucial role in synthesising surface structures that can be recognised by the immune system [41]. Additionally, both act as drug targets in diabetes and are essential for developing effective competitive inhibitors [42,43].

In the GO analysis of the strains Z45, the biological processes category encompassed the most cellular processes and metabolic processes. The molecular function category was most enriched for catalytic activity and binding (Figure 2E). KEGG annotation results showed that the highest number of genes of *L. plantarum* Z45 are involved in the carbohydrate metabolism pathway (17.30%, Figure 2F), followed by global and overview maps of cellular processes (13.67%) and amino acid metabolism (10.17%).

WGS revealed that *L. plantarum* Z45 possessed genes encoding various stress response proteins (Table 3). This aligns with the in vitro results that showed strain Z45 has acid and bile salt tolerance, strong gastrointestinal fluid tolerance, and antioxidant properties. The genome of *L. plantarum* Z45 contains ATP synthase, a Na^+^/H^+^ antiporter, and the ATP-dependent Clp protease ATP-binding subunit. These proteins facilitate extracellular H^+^ release and regulate pH, thereby enhancing the acid tolerance of the cells. This mechanism was consistent with the acid tolerance previously reported in *L. plantarum* [44]. Cyclopropane-fatty acyl-acyl phosphatidylcholine (CFP) synthetase and alkaline impact protein are key proteins for bile salt and acid resistance [45]. *L. plantarum* can withstand high temperatures owing to the presence of genes encoding heat stress-related proteins [46]. LAB biofilm formation is primarily associated with quorum sensing (QS) and the two-component system (TCS) of bacteria [47]. The TCS is a prevalent signal transduction mechanism in bacteria [48]. Z45 harbours genes encoding TCS-associated proteins, along with multiple genes associated with biofilm formation, such as *CpdA*, *TrpE*, *RpoN*, *CysE*, and *YidC*. LAB can resist oxidative stress by producing various antioxidant enzymes, inhibiting lipid peroxidation, chelating metal ions, neutralising free radicals, and modulating signalling pathways [49]. The whole-genome analysis of Z45 revealed it contains 5 genes encoding peroxide-related proteins, 14 genes encoding hydroxyl radical-related proteins, and 15 genes encoding oxidative stress-related proteins. As part of the cellular defense against oxidative stress, the msrC, msrA, and msrB genes encode methionine sulfoxide reductases. These enzymes are essential for protein maintenance under oxidative stress, as they detoxify reactive oxygen species by repairing the oxidative damage inflicted upon methionine residues within proteins [50]. Combined with the in vitro findings, these findings further corroborate the antioxidant properties of Z45. Therefore, fermenting foods with Z45 has the potential to scavenge free radicals and enhance the intestinal antioxidant defence system. The presence of these coding genes suggests that *L. plantarum* Z45 was equipped to endure various environmental stresses.

### 3.4. In Vitro Safety Evaluation

Haemolysis is a crucial factor to consider when assessing the safety qualities of probiotics, as haemolysins are exotoxins that can potentially lead to endocarditis and cell rupture, posing significant risks to human health [51]. Haemolysis assays showed that *L. plantarum* Z45 was negative for haemolytic activity (Supplementary Figure S2D,E). Furthermore, the absence of genes encoding haemolysin BL (*HbL*), non-haemolytic enterotoxin (*NhE*), and emetic toxin in the genome of *L. plantarum* Z45 is consistent with its lack of haemolytic activity. These results indicate that strain Z45 has a favourable safety profile.

Regarding the antibiotic resistance results for strain Z45 (Table 4), *L. plantarum* Z45 was resistance to gentamicin. To further validate the safety of the strain, we also conducted a minimum inhibitory concentration (MIC) test. The results indicated that the MIC of the strain which is below the threshold of 16 as per EFSA standards, demonstrating the absence of antibiotic resistance risk in strain Z45. However, *L. plantarum* Z45 was susceptible to penicillin, imipenem, minocycline, doxycycline, chloramphenicol, and clindamycin. Bacterial antibiotic resistance poses a significant risk to public health worldwide [52]. Some bacteria are intrinsically resistant, while others acquire resistance through mutation or horizontal transfer of resistance genes [53]. Lactobacillus generally has high resistance to aminoglycoside antibiotics [54]. It is attributed to their reduced membrane permeability to these antibiotics and is considered non-transferable since it is not mediated by acquired resistance genes [55,56]. Notably, the genome-wide analysis did not identify any antibiotic resistance genes, suggesting that strain Z45 is not at risk for horizontal transfer of antibiotic resistance genes. This genomic evidence, combined with the susceptible MIC phenotype, provides a dual assurance and strongly reinforces our conclusion that strain Z45 poses no foreseeable antibiotic resistance risk. In addition, it does not cause haemolysis or produce biogenic amines.

### 3.5. Secondary Metabolites and Comparative Genomes of Z45

AntiSMASH analysis predicted the bacteriocin production pathway. Strain Z45 was annotated with Type III polyketide synthases (*T3PKS*), terpene, and cyclic-lactone-autoinducer secondary metabolites, respectively (Figure 2G). *T3PKS* is a gene involved in encoding hydroxymethylglutaryl-CoA synthase and exhibits antimicrobial activity with potential as a biopreservative [57]. Terpenes have been reported to exhibit antimicrobial activity attributed to their ability to promote cell rupture and inhibit protein and DNA synthesis [58]. They are also considered excellent biocides in fermented foods [59].

### 3.6. Folate Metabolic Pathway of Z45

Folate deficiency results in significant disruptions in carbon metabolism, which may contribute to the onset of chronic illnesses and developmental problems. Mammalian cells cannot synthesise folic acid de novo; thus, dietary intake and supplements are the only sources of this essential nutrient [60]. Folic acid is essential for cellular growth and reproduction in organisms. *L. plantarum* Z45 was annotated as a producer of folic acid (Supplementary Figure S3). Specifically, 13 genes in the folate biosynthesis pathway and 11 genes associated with the one-carbon pool by the folate pathway were identified. These findings align with previously reported results on folic acid production by *L. plantarum* [61].

### 3.7. Experimental Results of Sensory Evaluation, Safety Assessment, and Physicochemical Parameters

Compared to sour soup fermented with other strains, the product fermented with strain Z45 showed improvements in appearance, odor, acidity, hardness, and overall score (Table 5). The Z45-fermented sour soup exhibited a smooth and regular surface, appropriate sourness with a slight aroma of corn, and a well-balanced taste profile characterized by moderate sweetness and sourness, medium firmness, slight stickiness, and good elasticity. In conclusion, the sensory properties of sour soup fermented with strain Z45 were superior to those of the other groups. The characteristic flavor of fermented foods has a significant impact on consumers’ purchasing decisions [62]. The fermentation quality of sour soup was significantly improved by using *L. plantarum* for fermentation. This finding is in line with earlier studies showing that LAB fermentation can modify the sensory properties of foods [63]. Testing of biogenic amine content in Z45 group fermentation samples indicates that the biogenic amine content in Z45 fermented sour soup is safe.

BAs are organic bases formed through microbial, plant, or animal metabolism, leading to the decarboxylation of amino acids. These compounds can be hazardous to human health [64]. Histamine and tyramine have been reported to exert toxic effects on vascular and psychoactive functions [65]. Putrescine and other polyamines play an important role in cell proliferation and have been linked to the occurrence of cancer [64]. The production of BAs by *L. plantarum* strains is rare, with only one strain previously identified as a BA-producing strain [65]. Moreover, *L. plantarum* strains have demonstrated the potential to degrade BAs in wine and fermented sausages, suggesting their utility as useful fermenters [66,67]. In addition, we assessed several physicochemical properties, including pH, total acidity, and total viable count (Table 6). The results demonstrated that fermentation with strain Z45 yielded superior performance compared to natural fermentation, which is also critical for the sensory evaluation and palatability of fermented foods [68,69].

### 3.8. Bacterial Community Composition

**Figure 3:**
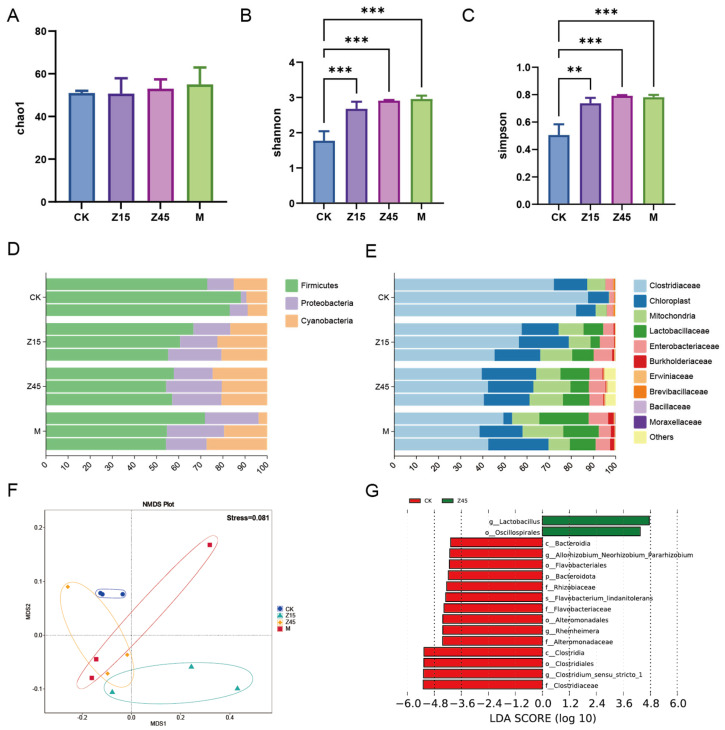
Results of the 16S rDNA of fermented sourdough seeds. (**A**) Chao 1; (**B**) Shannon index; (**C**) Simpson index; (**D**) Relative abundance of phylum level; (**E**) Relative abundance of genus level; (**F**) NMDS; and (**G**) LDA. ** indicate that *p* < 0.01 and *** indicate that *p* < 0.001, *n* = 3.

Food fermentation is initiated by a variety of microorganisms present in both major and minor components of the food mixture. Thus, the major nutrients are the primary determinants of the microbial community and metabolite composition [70]. Similarly, these components have varying effects on the nutritional and sensory properties and quality of the fermented product [71]. The Shannon and Simpson indices of the Z15, Z45 and M group were significantly higher than those of the traditionally fermented CK group (*p* < 0.05, Figure 3B,C), which may be attributed to probiotic supplementation, which enhances the measurement of diversity and abundance [72]. However, the difference in the Chao1 index was not significant (*p* > 0.05, Figure 3A). The analysis of microbial composition revealed that the predominant phyla were *Firmicutes*, *Proteobacteria*, and *Cyanobacteria* (Figure 3D). At the genus level, *Clostridiaceae*, chloroplasts, mitochondria, and *Lactobacillaceae* were the most abundant taxa (Figure 3E). Furthermore, an NMDS analysis of beta diversity demonstrated distinct clustering among the groups (Figure 3F). LEfSe analysis identified the order *Oscillospirales* and the genus *Lactobacillus* as significantly enriched biomarkers in the Z45 group. Generally, *Lactobacillus* spp. was significantly more abundant in the *L. plantarum* Z45 fermentation group compared with the CK group (*p* < 0.05, Figure 3G). *Lactobacillus* is regarded as the core microbiota of fermented corn and catalyses carbon metabolism [73]. *Oscillospirales* are recognized as a promising candidate for “next-generation” probiotics, capable of producing short-chain fatty acids such as butyrate, and have demonstrated beneficial effects in obesity-related metabolic disorders [74]. This property is particularly advantageous for conferring health benefits through fermented foods. It is noteworthy that the relative abundance of *Clostridium sensu stricto 1* was significantly lower in the Z45 group compared with the CK group. *Clostridium sensu stricto 1* is considered a pathogenic bacterium that causes inflammation, paediatric diarrhoea, and intestinal mucosal damage [75].

### 3.9. Non-Targeted Metabolome Analysis

**Figure 4:**
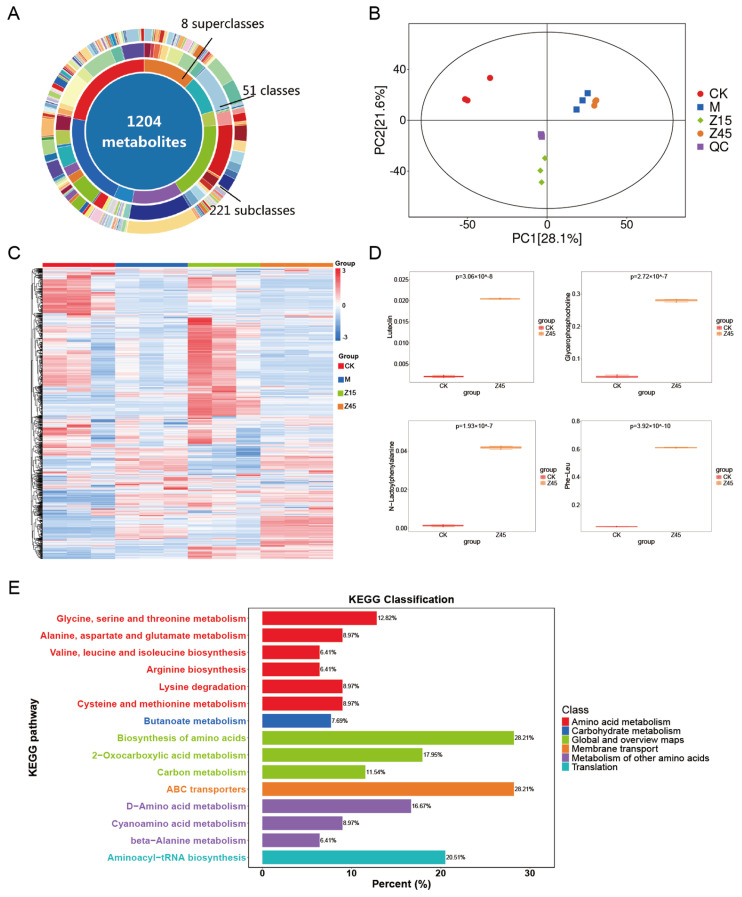
Results of the untargeted metabolome of fermented sourdough seeds. (**A**) Donut plot of metabolites; (**B**) OPLS-DA permutation; (**C**) TOTAL-heatmap; (**D**) Differential metabolites of CK vs. Z45; and (**E**) KEGG, *n* = 3.

Non-targeted metabolome analysis was employed to identify metabolite differences in naturally sour soup and those fermented with *L. plantarum* Z45. Initially, a total of 1204 metabolites were detected across the four groups (Figure 4A,C). An Orthogonal Partial least Squares–Discriminant Analysis (OPLS-DA) replacement test was conducted, and the regression line exhibited an upward trend, indicating that the replacement test was successful, and the model was not overfitted, resulting in two distinct clusters (Figure 4B). Luteolin, Glycerophosphocholine (GPC) and N-Lactoyl-Phenylalanine (Lac-Phe) were up-regulated in the Z45 group than in the traditionally fermented group (Figure 4D). Luteolin exhibits many biological activities, including anti-inflammatory, anti-allergic, and anti-cancer properties [76,77]. It also acts as a natural preservative [78] and aids in food preservation and the extension of shelf life [79]. GPC is a crucial precursor of the neurotransmitter acetylcholine and plays a vital role in brain and nervous system function [80]. It also modulates atherosclerosis, thereby reducing the risk of cardiovascular disease [81]. Lac-Phe has been identified as a taste-active amino acid in food fermentation, contributing to the enhancement of flavour in fermented foods [82]. In addition, it has potential anti-obesity effects [83]. KEGG signalling pathway annotations were predominantly associated with amino acid and carbohydrate metabolism (Figure 4E), suggesting that the incorporation of *L. plantarum* Z45 sourdough fermentation promotes the utilisation of amino acids and carbohydrates and may offer antioxidant, anti-obesity, and shelf-life extension benefits. Although this non-targeted metabolomics study has identified potential metabolites associated with obesity prevention and proposed the hypothesis that fermented foods might exert their effects through this pathway, these findings remain preliminary. To establish a definitive causal relationship, future studies should focus on employing targeted metabolomics approaches to conduct absolute quantification of the candidate metabolites, thereby accurately assessing their concentration changes in vivo and performing in vivo experiments using animal models to directly validate the efficacy and underlying mechanism of this compound in preventing obesity.

## 4. Conclusions

*L. plantarum* Z45 was isolated from sour soup, and exhibited a strong inhibitory effect on both Gram-positive and Gram-negative bacteria. Genomic analysis revealed that *L. plantarum* Z45 possesses a variety of probiotic genes, an abundance of carbohydrate-related genes, and several genes associated with folic acid production. Safety analyses validated that *L. plantarum* Z45 is non-haemolytic, does not produce harmful amines, lacks resistance genes, and poses minimal risk of becoming a human pathogen. Furthermore, three functional metabolites were identified in Z45. Metabolome analysis indicated that adding *L. plantarum* Z45 to fermented sourdough promotes amino acid and carbohydrate utilisation, and its metabolites exhibit antioxidant properties, obesity prevention potential, and shelf-life extension. Nevertheless, future in vivo studies are warranted to validate these findings. These findings suggest that *L. plantarum* Z45 is a safe and non-toxic strain with beneficial probiotic characteristics and resilience, which are essential for its survival and functionality in complex food production settings. Strain Z45 represents a promising candidate for starter cultures in fermented vegetable and soybean products, attributed to its robust fermentative capacity and potential role in flavor enhancement. In conclusion, the study expanded the application of *L. plantarum* in fermented food systems and offered new insights into enhancing the functionality of fermented foods.

## Figures and Tables

**Table 1:** Tolerance of isolated strain artificial gastrointestinal tract model.

Items	Simulated Gastric Fluid (logCFU/mL)		Survival (%)	Simulated Intestinal Fluid (logCFU/mL)		Survival (%)	Artificial Gastrointestinal Fluid (logCFU/mL)	Survival (%)
	0 h	3 h		0 h	3 h		6 h	
Z15	7.05 ± 0.10	6.17 ± 0.17 ^b^	87.45 ± 1.60 ^b^	8.02 ± 0.20	7.83 ± 0.14 ^a^	97.60 ± 0.72 ^a^	6.02 ± 0.12	85.43 ± 0.64
Z45	7.25 ± 0.14	7.06 ± 0.03 ^a^	97.37 ± 1.56 ^a^	8.08 ± 0.06	7.28 ± 0.04 ^b^	90.14 ± 0.17 ^b^	6.08 ± 0.03	83.87 ± 1.83

Note: Values are mean with SD of three replications. Different letters, ^a,b^, represent a significant difference between the two strains (*p* < 0.05).

**Table 2:** Prediction of strain virulence factors.

VFDB ID	Description	Gene	Virulence Factor
VFG000964 (gb|WP_010922799)	UTP-glucose-1-phosphate uridylyltransferase	*HasC*	Immune modulation
VFG002190 (gb|WP_002362225)	undecaprenyl diphosphate synthase	*Cpsa/Upps*	
VFG048830 (gb|WP_014907233)	NADP-dependent phosphogluconate dehydrogenase	*GndA*	
VFG046465 (gb|WP_003028672)	elongation factor Tu	*TufA*	Adherence
VFG012095 (gb|WP_003435012)	chaperonin GroEL	*GroEL*	
VFG037100 (gb|WP_010980745)	trifunctional thioredoxin/methionine sulfoxide reductase A/B protein	*Msra*	Stress
VFG000077 (gb|NP_465991)	ATP-dependent Clp protease proteolytic subunit	*ClpP*	
VFG000080 (gb|NP_464522)	ATP-dependent protease	*ClpE*	

**Table 3:** Probiotic potential genes of *L. plantarum* Z45.

Genes Detected in Z45	ID Number	Predicted Function
ATP synthase subunit a	00997	Acid tolerance
ATP synthase subunit b	00999	
ATP synthase subunit c	00998	
ATP synthase subunit alpha	01001	
ATP synthase subunit gamma	01002	
ATP synthase subunit beta	01003	
ATP synthase subunit epsilon	01004	
ATP synthase subunit delta	01000	
Na^+^/H^+^ antiporter napA	00663	
Na^+^/H^+^ antiporter nhaC	00168/02830	
Phosphotransferase system cellobiose-specific EIIB component (celA)	00673/01861	
L-lactate dehydrogenase	01212	
L-lactate permease	01495	
ATP-dependent Clp protease ATP-binding subunit ClpX	01242	
ATP-dependent Clp protease proteolytic subunit ClpP	01908	
ATP-dependent Clp protease ATP-binding subunit ClpE	02036	
ATP-dependent Clp protease ATP-binding subunit ClpC	02257	
Glucose-6-phosphate isomerase	00895	
Pyruvate kinase	01430	
Glucosamine-6-phosphate deaminase	00197	Acid/Bile tolerance
Cyclopropane-fatty-acyl-phospholipid synthase (CfA)	00407/01598	
Manganese-dependent inorganic pyrophosphatase (ppaC)	01478	
Alkaline shock protein	02335	
Putative universal stress protein	00498/00590/00707/00787/01022/01557/01593/01983/02081	
ABC-type transporter ATP-binding protein EcsA	01641/01812	
General stress protein (YugL)	01117	
Heat-inducible transcriptional repressor (HrcA)	01330	Heat shock defense
Molecular chaperone Hsp31 and glyoxalase (HchA)	00068	
Chaperone protein (DnaJ)	01333	
Chaperone protein (DnaK)	01332	
Chaperone protein (ClpB)	01425	
Molecular chaperone (GrpE)	01331	
Cold shock protein (Csp)	02145	
Cold shock protein (CspL)	00029	
Cold shock-like protein CspLA	02280	
ATP-dependent RNA helicase DeaD	02143	
DEAD-box ATP-dependent RNA helicase CshB	01075	
S-ribosylhomocysteine lyase luxS	02472	Biofilm formation
Catabolite control protein A *ccpA*	00146/01095	
Catabolite control protein B *ccpB*	00521/00820	
Family DNA-binding protein *ComEA*	01227	
DNA internalization-related competence protein ComEC	01229	
Biofilm regulatory protein A (BrpA)	00256	
Two-component system WalR/WalK regulatory protein (YycL)	00035	
Hydrogen peroxide-inducible genes activator OxyR	00543/00755/02182	Protection against peroxids
Organic hydroperoxide resistance transcriptional regulator (OhrR)	02368	
Peroxide operon regulator (PerR)	02758	
Putative oxidoreductase/MSMEI_2347	00113/00655/02869	Protection against Hydroxyl radicals
Putative oxidoreductase	00119/00615/01269	
Putative oxidoreductase (YghA)	00466	
Putative oxidoreductase (YhhX)	00816	
Putative oxidoreductase (YjmC)	02208	
Putative oxidoreductase (YceM)	02435	
Putative FAD-linked oxidoreductase	00257	
Quinone oxidoreductase (QorB)	02748/02751	
Oxidoreductase YdhF	02817	
Thiol peroxidase Tpx	01042	Oxidative stress
Thioredoxin TrxA	00205/01082/02904	
Thioredoxin-like protein YtpP	00798	
Thioredoxin reductase TrxB	02483	
Hsp33 family molecular chaperone HslO	02676	
I-methionine (R)-S-oxide reductase MsrC	01037	
Peptide-methionine (S)-S-oxide reductase MsrA	01354/01480/01969	
Peptide-methionine (R)-S-oxide reductase MsrB	01479	
NADH peroxidase NpR	00858/01822	
NADH oxidase NoX	01387/02478/02484/02914	
Elongation factor Tu (TuF)	01240	Adhesion and aggregation
Pyruvate dehydrogenase El component beta (pdhB)	01209	
Enolase (eno)	01408/01410/02456	
Glyceraldehyde-3-phosphate dehydrogenase GapA	02459	
Triosephosphate isomerase TpiA	02457	

**Table 4:** Antibiotic resistance of *L. plantarum* Z45.

Antimicrobial Classes	Antimicrobial Agents	Disk Dose (μg)	Inhibition Zone Diameters/mm (IZD) ^a^
β-lactams antibiotics	Penicillin	10	S
	Ampicillin	10	I
	Imipenem	10	S
Aminoglycosides antibiotics	Gentamicin	10	R
Tetracyclines	Minocycline	30	S
	Doxycycline	30	S
	Chloramphenicol	30	S

^a^: R, Resistant (≤15 mm); I, Intermediate (16–20 mm); S, Susceptible (≥21 mm).

**Table 5:** Results of sour soup sensory evaluation.

Items	Appearance	Odor	Acidity	Hardness	Viscosity	Elasticity	Overall Score
CK	10.75 ± 1.86 ^a^	13.92 ± 1.62 ^a^	21.42 ± 2.54 ^a^	7.33 ± 0.89 ^a^	7.50 ± 0.80	12.17 ± 1.11	73.08 ± 4.29 ^a^
Z15	11.92 ± 1.00 ^b^	14.83 ± 2.48 ^ab^	21.50 ± 2.32 ^a^	7.50 ± 0.80 ^a^	7.75 ± 1.06	12.75 ± 0.97	76.25 ± 4.63 ^b^
Z45	13.42 ± 0.90 ^c^	16.25 ± 1.29 ^b^	23.67 ± 1.30 ^b^	8.42 ± 0.79 ^b^	8.33 ± 0.89	13.08 ± 1.08	83.17 ± 3.04 ^c^
M	11.92 ± 1.00 ^b^	15.08 ± 0.67 ^ab^	22.33 ± 1.23 ^ab^	7.92 ± 0.67 ^ab^	7.83 ± 0.58	12.58 ± 1.00	77.67 ± 2.50 ^b^

Note: Different letters within a column indicate significant differences (*p* < 0.05).

**Table 6:** Results of sour soup physicochemical parameters and biogenic amine content.

Items	Content			
	CK	Z15	Z45	M
pH	3.87 ± 0.05 ^a^	3.80 ± 0.04 ^ab^	3.70 ± 0.09 ^b^	3.83 ± 0.03 ^a^
TA (g/L)	10.33 ± 0.50 ^a^	11.70 ± 0.56 ^b^	12.37 ± 0.55 ^b^	11.53 ± 0.50 ^b^
Viable count (log CFU/mL)	7.57 ± 0.25 ^a^	7.99 ± 0.18 ^b^	8.15 ± 0.13 ^b^	8.03 ± 0.21 ^b^
Putrescine (mg/kg)	17.05 ± 1.88 ^a^	12.04 ± 1.70 ^b^	4.12 ± 0.63 ^d^	8.23 ± 0.21 ^c^
Tryptamine (mg/kg)	17.87 ± 1.11 ^a^	16.51 ± 1.31 ^a^	2.99 ± 0.22 ^c^	11.11 ± 1.17 ^b^
Phenylethylamine (mg/kg)	5.31 ± 0.54 ^a^	0.48 ± 0.07 ^b^	0.24 ± 0.04 ^b^	0.42 ± 0.07 ^b^
Cadaverine (mg/kg)	8.55 ± 1.04 ^a^	0.51 ± 0.05 ^b^	0.45 ± 0.08 ^b^	0.45 ± 0.07 ^b^
Histamine (mg/kg)	3.94 ± 0.36 ^a^	3.80 ± 0.31 ^a^	0.26 ± 0.08 ^c^	0.26 ± 0.08 ^b^
Tyramine (mg/kg)	4.73 ± 0.59 ^a^	4.25 ± 0.90 ^a^	0.45 ± 0.09 ^c^	2.70 ± 0.46 ^b^
Spermidine (mg/kg)	0.28 ± 0.07	0.26 ± 0.05	0.16 ± 0.04	2.73 ± 0.32
Spermine (mg/kg)	0.13 ± 0.05 ^ab^	0.21 ± 0.05 ^a^	0.08 ± 0.03 ^b^	0.16 ± 0.05 ^ab^

Note: Different letters within a column indicate significant differences (*p* < 0.05).

## Data Availability

The original contributions presented in the study are included in the article/Supplementary Materials, further inquiries can be directed to the corresponding author.

## Supplementary Materials

The following supporting information can be downloaded at: https://www.mdpi.com/article/10.3390/foods14213803/s1, Figure S1: Antibacterial heatmap and antioxidation of strain. (A) Antibacterial heatmap; (B) DPPH scavenging rate; (C) ABTS scavenging rate; and (D) Superoxide anion radical scavenging activity; Figure S2. Morphology, staining and phylogenetic tree of *Lactobacillus plantarum* Z45. (A) morphology; (B) Gram staining; (C) Electron microscopy; (D) and (E) Blood plate hemolysis results of Z45; Figure S3. Folic acid signaling pathway annotations of *Lactobacillus plantarum* Z45; Table S1: Genome Database of *Lactobacillus plantarum* Z45; Table S2. Sensory Evaluation of Sour Soup; Table S3. General genome features of *Lactobacillus plantarum* Z45; Table S4. Prediction of insertion sequence in *Lactobacillus plantarum* Z45; Table S5. Prediction of prophage in *Lactobacillus plantarum* Z45.
