# Supplementary material for: Lactiplantibacillus plantarum Z45 from Sour Soup Improves Flavor and Safety of Fermented Corn: Insights from Genomic and Metabolomic Approaches
Source: Foods. 2025 Nov 6;14(21):3803. doi: 10.3390/foods14213803 (PMC12609953; doi:10.3390/foods14213803)
Supplement: Supplementary file 1 [file foods-14-03803-s001.zip › foods-3924510-supplementary.pdf]

Lactobacillus plantarum Z45 from sour soup improves flavor and safety of fermented corn: Insights from genomic and metabolomic approaches

Mengdi ZHAO [1,2], Yuanyuan ZHANG [1], Yi WU[1], Yueyao LI [1], Shuang LIANG[1], and Guangyu LI [1*]

1 College of Animal Science and Technology, Qingdao Agricultural University, Qingdao, 266109, China

2 College of Animal Science and Technology, Shandong Agricultural University, Taian, 271018, China

*Correspondence: Guangyu Li (tcslgy@126.com)

Tel: +86-18043213500. Fax: +86-37-58957722.

Table S1. Genome Database of *Lactobacillus plantarum Z45*.

Database	Description or version	Homepage
NR	non-redundant protein sequence database	ftp://ftp.ncbi.nih.gov/blast/db/
GO	Gene Ontology database	http://www.geneontology.org/
COG	The database of Clusters of Orthologous Groups of proteins	http://www.ncbi.nlm.nih.gov/COG/
KEGG	The database of Kyoto Encyclopedia of Genes and Genomes	http://www.genome.jp/kegg/
Prokka	Rapid prokaryotic genome annotation (1.14.6)	https://github.com/tseemann/prokka
BLAST+	Basic Local Alignment Search Tool (2.11.0+)	https://blast.ncbi.nlm.nih.gov/Blast.cgi
CAZy	2021-09-24	http://www.cazy.org/
PHI	4.12	http://www.phi-base.org/
CARD	3.2.0	http://arpcard.mcmaster.ca
VFDB	2022-03-11	http://www.mgc.ac.cn/VFs/
Pseudofinder	v1.1.0	https://github.com/filip-husnik/pseudofinder
antiSMASH	7.0	https://docs.antismash.secondarymetabolites.org/
CheckM	v1.1.6	http://ecogenomics.github.io/CheckM/

Table S2. Sensory Evaluation of Sour Soup

Item	Sensory Indicator	Score
**Appearance (15)**	Smooth and regular	13–15
	Relatively smooth and regular	10–12
	Slightly rough	7–9
	Mildly deformed	4–6
	Severely deformed	0–3
**Odor (20)**	Moderately sour with a distinct corn aroma	17–20
	Appropriately sour with a slight corn aroma	13–16
	Too weak or too strong in sourness, with a faint corn aroma	9–12
	Too weak or too strong in sourness, with a slightly pungent odor	5–8
	Significant off-odor or strong unpleasant smell	0–4
**Acidity (30)**	Pleasant taste, well-balanced sweet and sour flavor	25–30
	Rel pleasant, fairly balanced sweet and sour taste	19–24
	Too mild or too sour	13–18
	Poor sweet-sour balance but without off-flavors	7–12
	Unpleasant taste, severely imbalanced or with off-	0–6

Item	Sensory Indicator	Score
	flavors	
**Hardness (10)**	Moderately firm	9–10
	Slightly too hard or too soft	7–8
	somewhat hard or soft	5–6
	Too hard or too soft	3–4
	No firmness perceived	0–2
**Viscosity (10)**	Smooth and refreshing with no stickiness	9–10
	Slightly sticky	7–8
	somewhat sticky	5–6
	Sticky	3–4
	Extremely sticky and unpleasant	0–2
**Elasticity (15)**	Highly elastic and springs back completely	13–15
	Fairly elastic	10–12
	Moderately elastic	7–9
	Poor elasticity	4–6
	No elasticity, does not recover when pressed	0–3

Table S3. General genome features of *Lactobacillus plantarum Z45*.

Indicator	Z45
Genome Size (Mbp)	3.05
Plasmid	14
G+C content (%)	44.89
Coding gene numbers	2,932
Total length of coding genes (bp)	2,595,830
Gene/Geonme (%)	85
rRNAs	4
tRNA	9
tRNA genes	69
tmRNA	1
Sequencing Depth	333
Coverage	176
Scaffold N50	3,046,868
number of contig	15
Completeness (%)	99.38

Table S4. Prediction of insertion sequence in *Lactobacillus plantarum* Z45.

Transposon	Identity(%)	Length	Mismatches	q.start	g.end	e-value	Bit score
ISP2	98.89	1796	20	2532646	2534441	0.0	3402
ISP2	98.89	1796	20	564433	566228	0.0	3402
ISP2	98.89	1796	20	2316709	2318504	0.0	3402
ISP2	98.89	1796	20	1622470	1624265	0.0	3402
ISP2	98.89	1796	20	403712	405507	0.0	3402
ISP2	98.89	1796	20	2649606	2651401	0.0	3402
ISP2	98.89	1796	20	2739725	2741520	0.0	3402
ISP2	98.83	1796	21	151916	153711	0.0	3394
ISP2	91.84	49	4	881578	881626	4e-07	65.9
ISP2	92.86	42	3	151948	151989	2e-05	60.0
ISP2	92.86	42	3	2741447	2741488	2e-05	60.0
ISP2	92.86	42	3	2651328	2651369	2e-05	60.0
ISP2	92.86	42	3	403744	403785	2e-05	60.0
ISP2	92.86	42	3	1622502	1622543	2e-05	60.0
ISP2	92.86	42	3	2316741	2316782	2e-05	60.0
ISP2	92.86	42	3	566155	566196	2e-05	60.0
ISP2	92.86	42	3	2532678	2532719	2e-05	60.0
ISLpl2	99.73	1491	4	554498	555988	0.0	2924
ISLpl2	99.73	1491	4	2268947	2270437	0.0	2924
ISLpl2	99.73	1491	4	1178972	1180462	0.0	2924
ISLpl2	99.60	1491	6	1566799	1568289	0.0	2908
ISLpl2	94.59	37	2	1312384	1312420	9e-05	58.0
ISLpl2	82.61	92	16	2853583	2853674	4e-04	56.0
IS1310	95.86	1352	56	427180	428531	0.0	2236
IS1310	95.86	1352	56	1968642	1969993	0.0	2236
IS1310	95.86	1352	56	725935	727286	0.0	2236
IS1310	95.78	1352	57	2046756	2048107	0.0	2228
ISLpl1	99.04	1043	10	266850	267892	0.0	1988
ISLpl1	98.95	1043	11	471101	472143	0.0	1980
ISPp1	97.99	1043	17	266850	267892	0.0	1869
ISPp1	97.89	1043	18	471101	472143	0.0	1861
ISLpl3	99.77	853	1	2995831	2996683	0.0	1667
ISLpl3	99.77	853	1	136764	137616	0.0	1667
ISLpl3	99.77	853	1	372706	373558	0.0	1667
ISLpl3	99.77	853	1	2400310	2401162	0.0	1667
ISLpl3	99.77	853	1	1258196	1259048	0.0	1667
ISLpl3	99.77	853	1	1269978	1270830	0.0	1667
ISLpl3	99.77	853	1	932560	933412	0.0	1667
ISLpl3	99.77	853	1	440786	441638	0.0	1667
ISLpl3	99.77	853	1	2794658	2795510	0.0	1667
ISLpl3	99.77	853	1	165422	166274	0.0	1667
ISLpl3	99.77	853	1	2925260	2926112	0.0	1667
ISLpl3	99.65	853	2	778290	779142	0.0	1659
ISLpl3	99.53	853	3	840369	841221	0.0	1651
IS153	97.82	596	13	360195	360790	0.0	1078
IS153	97.48	596	15	2364872	2365467	0.0	1063
IS153	96.98	596	18	962423	963018	0.0	1039
IS153	95.20	542	25	961668	962208	0.0	860
IS153	94.99	539	26	361008	361545	0.0	846
IS153	94.81	539	27	2365685	2366222	0.0	839
ISLsa1	85.23	1036	150	2627732	2628767	0.0	827
ISLhe30	86.72	128	15	1424364	1424491	2e-18	103

ISLhe30	80.53	226	44	1423481	1423706	3e-17	99.6
ISLhe30	86.60	97	13	1423855	1423951	3e-14	89.7
ISLhe30	86.67	60	8	1424095	1424154	4e-04	56.0
KIS	86.96	92	12	2094385	2094476	1e-13	87.7
ISBame1	96.97	33	1	427180	427212	9e-05	58.0
ISBame1	96.97	33	1	1969961	1969993	9e-05	58.0
ISBame1	96.97	33	1	2046756	2046788	9e-05	58.0
ISBame1	96.97	33	1	727254	727286	9e-05	58.0

Table S5. Prediction of prophage in *Lactobacillus plantarum* Z45.

PP_start	PP_end	attL_start	attL_end	attR_start	attR_end	attL_sequence	attR_sequence
2616727	2644240	2618524	2618537	2644900	2644913	GGCGGTCGTCAAT	GGCGGTCGTCAAT

PP_start: Start position of the prophage on the contig; PP_end: End position of the prophage on the contig; attL_start: Start position of attL; attL_end: End position of attL; attR_start: Start position of attR; attR_end: End position of attR; attL_sequence: Nucleotide sequence of attL; attR_sequence: Nucleotide sequence of attR.

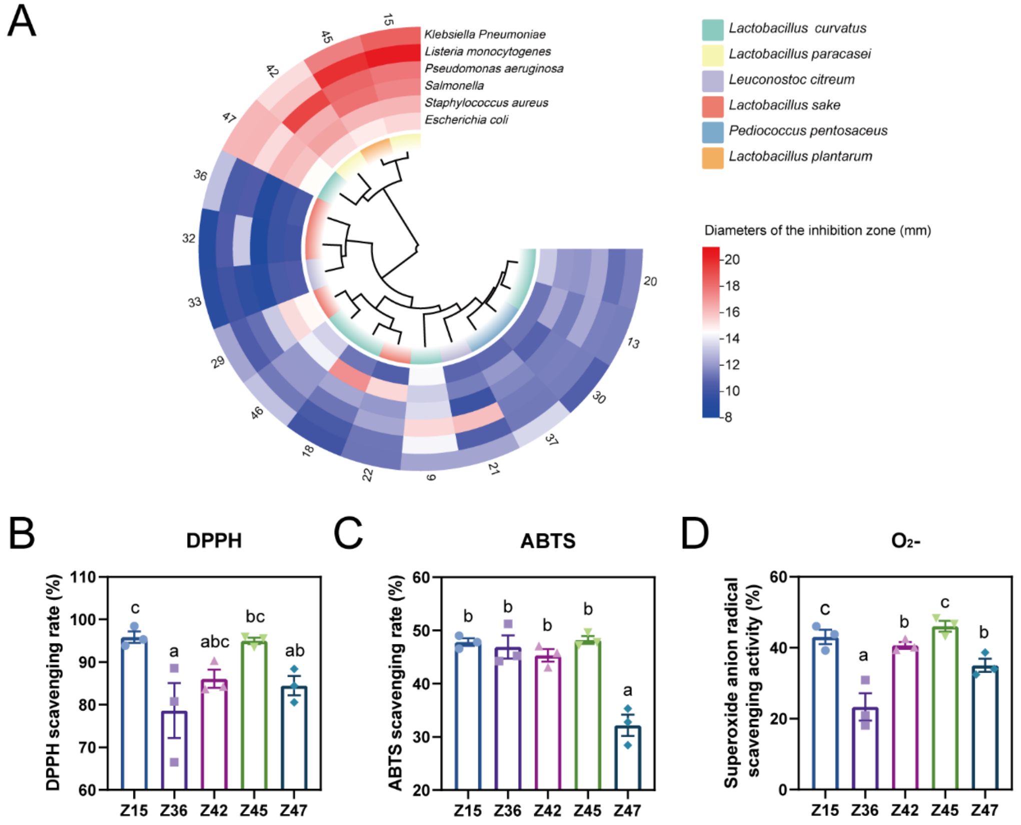

Figure S1. Antibacterial heatmap and antioxidation of strain. (A) Antibacterial heatmap; (B) DPPH scavenging rate; (C) ABTS scavenging rate; and (D) Superoxide anion radical scavenging activity.

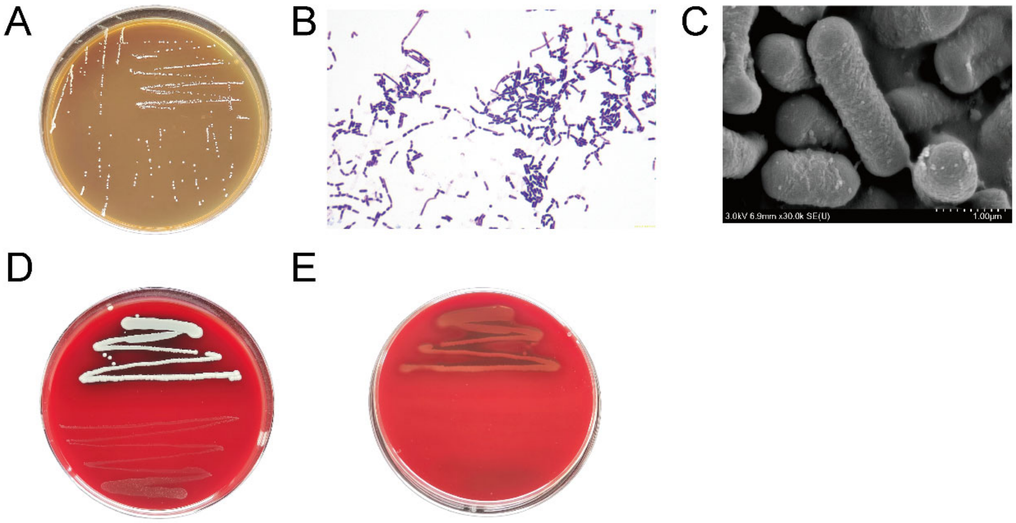

Figure S2. Morphology, staining and phylogenetic tree of *Lactobacillus plantarum* Z45. A) morphology; B) gram staining; C) Electron microscopy; D) and E) Blood plate hemolysis results of Z45.

A

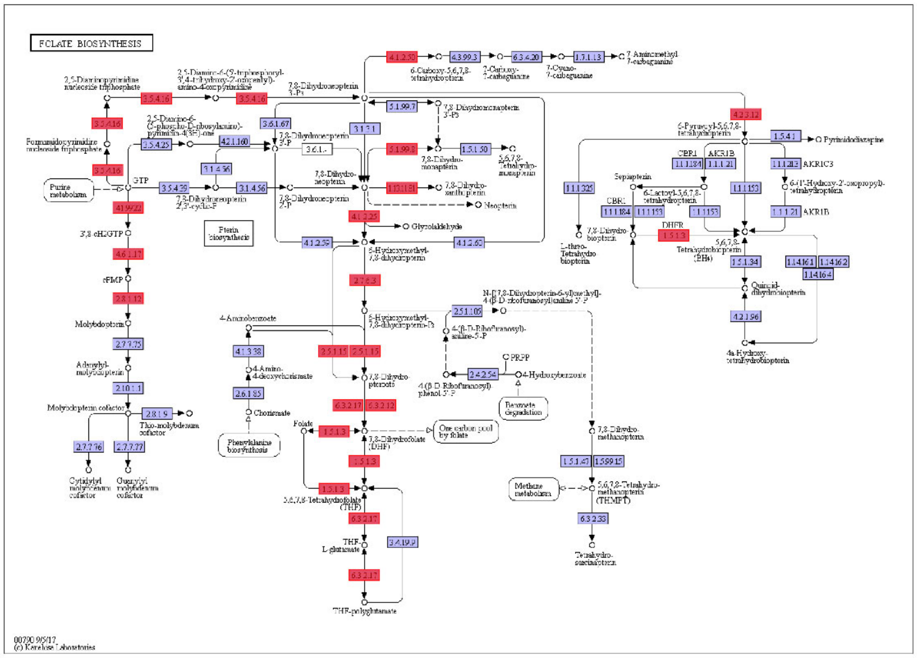

B

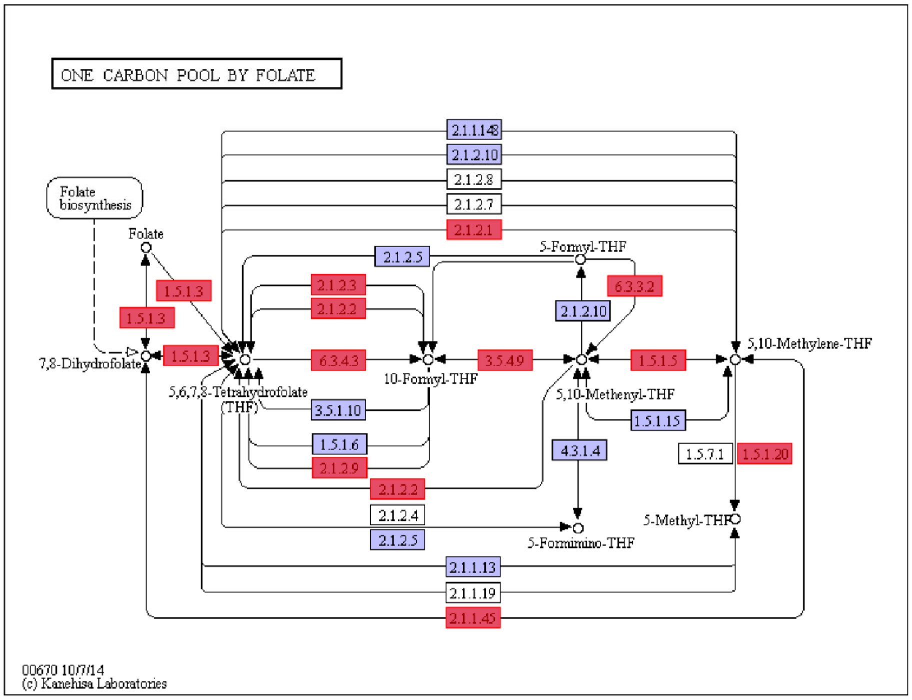

Figure S3. Folic acid signaling pathway annotations of *Lactobacillus plantarum* Z45.
